# Prediction and Experimental Evaluation of Mechanical Properties of SiC-Reinforced Ti-4.25Al-2V Matrix Composites Produced by Laser Direct Energy Deposition

**DOI:** 10.3390/ma16155233

**Published:** 2023-07-25

**Authors:** Ilya Magidov, Konstanitin Mikhaylovskiy, Svetlana Shalnova, Ilya Topalov, Marina Gushchina, Sergey Zherebtsov, Olga Klimova-Korsmik

**Affiliations:** 1 Department of Aerospace Composite Structures, Bauman Moscow State Technical University, 105005 Moscow, Russia; j-bright@mail.ru (I.M.); mikhaylovskiy@bmstu.ru (K.M.); 2 World-Class Research Center “Advanced Digital Technologies”, State Marine Technical University, 190121 Saint Petersburg, Russia; s.shalnova@corp.smtu.ru (S.S.); ilya_topalov@mail.ru (I.T.); gushcina_mo@corp.smtu.ru (M.G.); o.klimova@ltc.ru (O.K.-K.); 3 Mathematics and Mechanics Faculty, Saint Petersburg State University, 198504 Saint Petersburg, Russia; 4 Laboratory of Bulk Nanostructured Materials, Belgorod National Research University, 308015 Belgorod, Russia

**Keywords:** laser direct energy deposition (LDED), titanium alloy, particle-reinforced composite materials (PRCM), composite materials

## Abstract

An important direction in the development of additive technologies is associated with the addition of ceramic particles (oxide, carbide, boride, and nitride ceramics) to metal powders. The prediction of the physical and mechanical characteristics of SiC-particle-reinforced composite materials (PRCMs) in comparison with experimental results was studied. A near-α Ti-4.25Al-2V titanium-alloy-based composite reinforced by 1 vol.% of SiC ceramic particles was produced using laser direct energy deposition. A multiscale modeling approach at the micro and macro levels was applied. At the micro level, the toughness and strength characteristics for a temperature interval of T = 20–450 °C were predicted using a representative volume element of PRCM with the nearly real shape of SiC particles. At the macro level, the features of plastic deformation and fracture of the PRCM were predicted by numerical modeling using the commercial software Digimat Student Edition ver. 2022.4 and Ansys Student 2023 R2. The addition of SiC particles was found to improve the physical and mechanical properties in the whole temperature range. The results of the numerical modeling were consistent with the experimental data (the deviation did not exceed 10%). The proposed approach for predicting the physical and mechanical properties of Ti-4.25Al-2V/SiC can also be used for other PRCMs obtained by laser direct energy deposition.

## 1. Introduction

Additive technologies (ATs) have become an important tool in various industries, including the aircraft and space industry, medical technology, automotive applications, and many others [1,2,3,4,5,6]. One of their benefits is associated with the ability to produce complex geometric shapes, which are sometimes impossible to achieve using traditional manufacturing methods [7,8]. Moreover, new materials with unique structures and compositions can be produced by ATs [9,10,11]. Specifically, particle-reinforced composite materials (PRCMs) based on titanium alloys with improved mechanical properties [12,13] can be obtained using laser direct energy deposition (LDED).

In this case, particles of refractory ceramic materials, such as carbides [14,15], borides [16], or nitrides [17,18], are added to the matrix alloy powder to improve its mechanical characteristics. However, this approach also has some limitations. For example, the heterogeneous distribution of the particles can result in some non-uniformity in the structure and properties of the material, and residual stresses arising during LDED can cause local areas with an increased tendency to cracking, particularly in the presence of brittle particles. For example, P. Krakhmalev et al. [19] present a study on the fabrication of multiphase composite coatings with an ultrafine microstructure using the selective laser melting of Ti and (20, 30, 40 wt.%)SiC powder mixtures. The coatings were found to contain TiCx, Ti_5_Si_3_Cx, TiSi_2_, and SiC phases and exhibited high hardness and abrasion resistance properties, making them promising for applications involving abrasive wear. The study also identified two types of structural inhomogeneity in the coatings and proposed an increase in laser power as a way to enhance the homogeneity of the coatings, although further refinement of the process parameters is still required for the fabrication of fully dense coatings. Neng Li et al. [20] obtained the same results during the laser cladding process; chemical reactions took place between Ti and SiC particles in the composite layers, and the resulting reaction products consisted of TiC and Ti_5_Si_3_. The thermodynamic analysis showed that the reactions between Ti and SiC could occur spontaneously at high temperatures. The addition of SiC increased the microhardness of the coatings due to the formation of hard intermetallic compounds such as TiC and Ti_5_Si_3_. The flexure test showed an improvement in the mechanical strength of the coatings with higher SiC concentrations.

Nevertheless, in general PRCMs are expected to possess more balanced characteristics in comparison with “usual” metallic materials. The majority of particle-reinforced composite materials are generated by the vacuum sintering method [21,22]. After sintering, the differences in the physical and chemical properties between the cores and rims have led to more complex interfacial stress distribution on the different interfaces of the composite materials; thus, the ability of the materials to resist crack propagation is reduced. However, the traditional experimental methods for selecting new materials are usually labor-intensive, so the development of predictive approaches is becoming increasingly attractive [23]. In spite of some difficulties caused, for example, by the possibility of chemical interactions between the metal matrix and ceramic inclusions, there have been many successful attempts at predicting the physical and mechanical characteristics of composite materials. The article [24] outlines a study of the mechanical behavior of nanocomposite materials, which includes interfaces, using both experimental and numerical methods. The methods for calculating the mechanical properties of nanoelement-reinforced composites are discussed, and a multiscale model is presented for calculating elastic constants and local/interface properties for systems with statistically homogeneous distributions of embedded nanoelements. However, the article did not mention the possible limitations of the research methods used, nor did it provide details on how the materials for the experimental and numerical methods were chosen. Ferdi Yıldırım et al. [25] discuss the use of numerical computational techniques in estimating the physical and mechanical properties of polymeric composites, with a focus on the multiscale modeling technique. The finite element method was applied for the numerical modeling of PRCM using the Digimat-FE module with further experimental verification. The results of this study also suggest a very good correlation of the prediction and experimental data. The representative volume element is used to model composite materials, and structural tests are simulated to predict mechanical properties. The mean-field homogenization method is used at the microscale level to obtain optimal mechanical properties, and the mesoscale method is used at the structural level to achieve a homogenous material model. The Digimat Student Edition ver. 2022.4 is used to predict the nonlinear behavior of multiphase materials. However, it should be noted that the numerical modeling of PRCMs at the macro level usually ignores the effect of the sample’s geometry.

As regards the study of the formation of PRCMs, previous studies have focused on microstructural effects and phase transformations, avoiding the topics of the mathematical modeling and validation of mechanical properties at elevated temperatures. A survey of the available scientific literature showed a lack of reliable data on the development of numerical models and their experimental confirmation with respect to PRCMs based on metallic materials, especially the near-α titanium alloy +1 vol.% SiC obtained by the LDED process. The present study focused on a novel approach to predicting the physical and mechanical properties of PRCMs. The study used a multiscale modeling approach with the Digimat Student Edition ver. 2022.4 and Ansys Student 2023 R2 software to predict the behavior of the composite materials under various loads, followed by experimental verification of the obtained numerical results. The addition of SiC particles was found to improve the physical and mechanical properties in the whole temperature range, and the proposed numerical modeling approach can be used for other PRCMs obtained by the LDED method.

## 2. Materials and Methods

### 2.1. Description of Calculation Methods

In the present study, the Digimat Student Edition ver. 2022.4 with the “Digimat-FE” module (MSC Software, Newport Beach, California, USA) was used for the prediction of the physical–thermal–mechanical properties of the PRCM [26,27]. The following parameters and assumptions were used:

— No porosity and cracking, and crack formation and propagation was not considered;
— Volume fraction of reinforcements—1%;
— Particle size—30–90 μm;
— Calculating volume—1.45 × 1.45 × 1.45 mm^3^;
— A flake-like shape of the particles;
— The bond between particles and matrix was ideal without any defects;
— The numerical model for the tensile sample was in accordance with ASTM E21-20 [28];
— The load was applied in accordance with that used in the experiment.

The Ansys Student 2023 R2 (Ansys Inc., Canonsburg, PA, USA), in particular AUTODYN and Ansys Explicit Dynamics module, was used for the numerical modeling of the physical processes and strength of LDED. The following samples and assumptions were used:

— The samples measured ∅4 × 20 mm as per ASTM E21-20 for static tests. The finite element size was 0.5 mm.
— One side of the sample was fixed while the total displacement and speed of another side was 10 mm and 0.1 mm/sec, respectively.

### 2.2. Materials and Equipment

**Figure 1:**
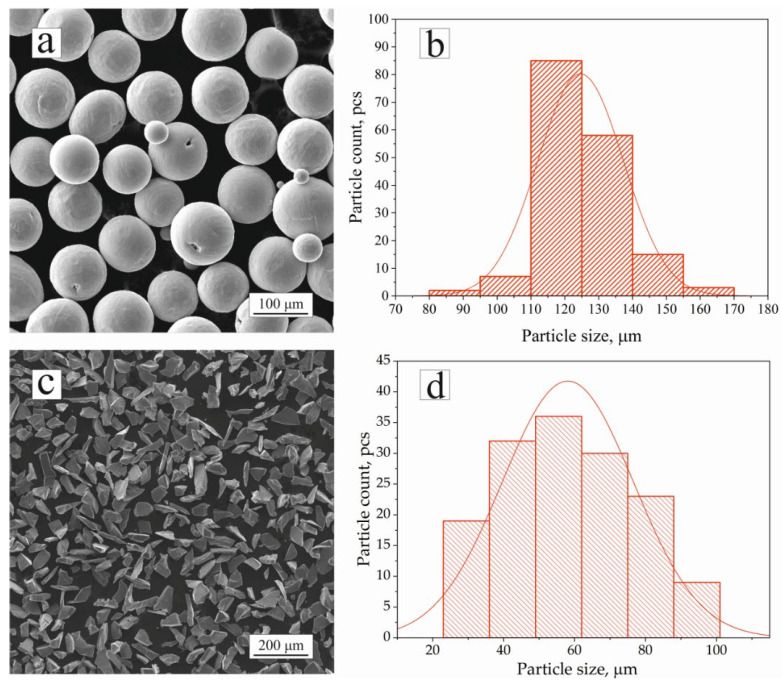
SEM images of Ti-4.25Al-2V (**a**) and SiC (**c**) powder particles; particle size distribution of Ti-4.25Al-2V (**b**) and SiC (**d**).

Near-α titanium Ti-4.25Al-2V alloy powder (LLC “GK SMM”, Moscow, Russia) and ceramic silicon carbide (SiC) powder (LLC “Sapphire”, Moscow, Russia) were used for the production of the PRCM samples. The chemical composition of the powders was determined using a Mira3 (Tescan Orsay Holding, a.s., Brno, Czech Republic) scanning electron microscope (SEM) equipped with an energy-dispersive X-ray spectroscopy (EDX) unit Ultim Max 65 (Oxford Instruments NanoAnalysis, Abingdon, United Kingdom). The measured chemical composition in wt.% of the titanium alloy powder was 93.9 Ti, 4.2Al, 1.9 V, and the SiC powder was 69.7 Si, 30.3 C. The fractional composition of the powder and width of the transition zone were measured using Digimizer image analysis software 6.3.0 (MedCalc Software Ltd., Ostend, Belgium). The titanium powder had a spherical shape with an average diameter of 125 µm (Figure 1a,b); the SiC powder had an average particle size of 54 µm and an irregular pyramidal shape (Figure 1c,d).

**Figure 2:**
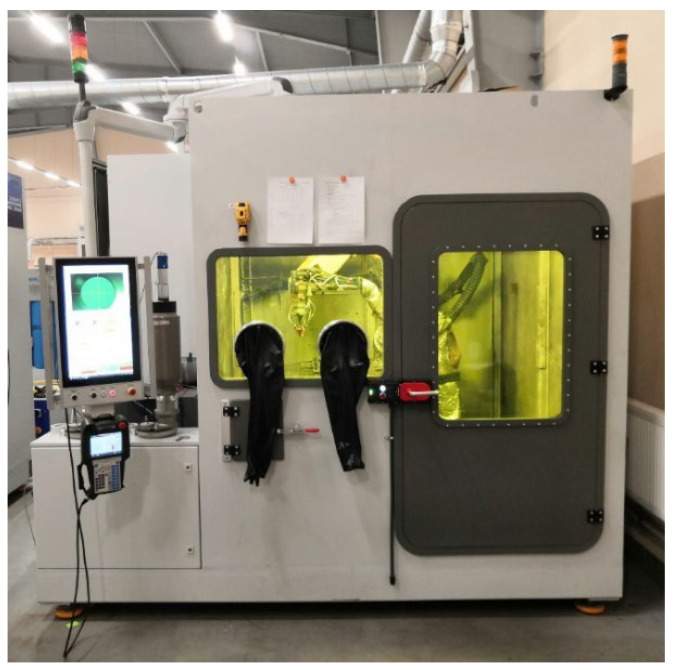
ILIST-M robotic complex for laser direct energy deposition.

The production of the samples was carried out using a robotic complex (Figure 2) for laser direct energy deposition, ILIST-M (Saint Petersburg State Marine Technical University, Saint Petersburg, Russia) [29]. The complex included a fiber laser, with a maximum power of 3 kW, a 6-axis robotic manipulator and a 2-axis tilt-and-turn positioner, and a sealed cabin of 9 m^3^ filled with protective gas (argon) with a residual oxygen level below 100 ppm. Titanium is a chemically active element and in the molten state is able to quickly react with the environment. Argon gas is an inert protective gas and does not interact with molten metal, which prevents the formation of brittle compounds, such as titanium oxide or nitride; their presence in the metal greatly reduces the mechanical properties of materials. The working tool was a laser welding head with a four-ray nozzle with a local supply of argon into the molten zone. 

To ensure the required chemical composition of the PRCM, Ti-4.25Al-2V and SiC powders were simultaneously fed in the proper ratio from 2 feeders into the molten pool. The technological parameters are given in Table 1. In order to relieve residual stresses, the LDED samples were then soaked at 860 °C for 30 min in a SNOL 30/1200 muffle furnace (AB Umega-Group, Lithuania) and then cooled in the air.

Uniaxial tensile tests of the LDED samples, produced as per ASTM E21-20, were carried out at 23 °C, 250 °C, 350 °C, and 450 °C in the air using Zwick/Roell (Zwick Roell Group, Ulm, Germany) testing machines. Two samples were tested at each temperature. Heating to the target temperature was performed using a circular three-zone high-temperature furnace (LLC “Metrotest”, Moscow, Russia) at a rate of 30 °C/min. The fracture surfaces were investigated using a Tescan scanning electron microscope (SEM) equipped with a secondary electron detector (SE).

## 3. Results and Discussion

### 3.1. Numerical Modeling of Properties of PRCM

**Figure 3:**
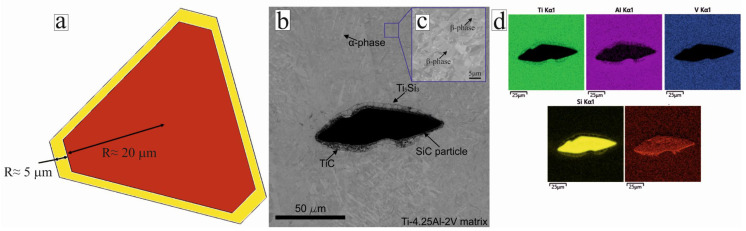
Model image of reinforcing SiC particles (denoted by red) and the predicted transition zone of chemical interaction with the Ti-4.25Al-2V matrix (denoted by yellow) (**a**); microstructure of the composite (**b**); microstructure at 3000 magnification (**c**); distribution of chemical elements in the transition zone between SiC and Ti-4.25Al-2V matrix (**d**).

The numerical modeling of the physical and mechanical characteristics of the PRCM was performed using the Digimat-FE module. To improve the accuracy of the prediction, the transition zone between the particle and the matrix was also considered (Figure 3) by the Digimat-FE module. This zone can influence the matrix-to-particle load transfer, thereby affecting the overall strength and deformation characteristics of the material. This approach allowed us to evaluate the efficiency of the load transfer and to determine the effect of the structural parameters on the mechanical properties of the composite. Additionally, stress concentrations often occur in the transition zone due to the different properties of the matrix and particles. The transition zone modeling allowed us to evaluate these stress concentrations and to predict possible locations of failure. 

The predicted width of the transition zone was 5–10 µm; this was also confirmed by the results of measuring the transition zone on the SEM images. The actual thickness was 4–11 µm (Figure 3b). The microstructure of the Ti-4.25Al-2V matrix consisted mainly of α-lamellae with a small amount of the β-phase (Figure 3c). Chemical analysis suggested that the transition zone around the SiC particles comprised TiC and Ti_5_Si_3_ phases (Figure 3d). The same phases were found in [30] using various methods. Following the interfacing reaction, TiC and Ti5Si3 phases are formed by the diffusion process [31]. Furthermore, according to [20], chemical reactions occur between Ti and SiC particles in the composite layers during the laser cladding process at the microstructure level, resulting in the formation of reaction products such as TiC and Ti5Si_3_.

**Figure 4:**
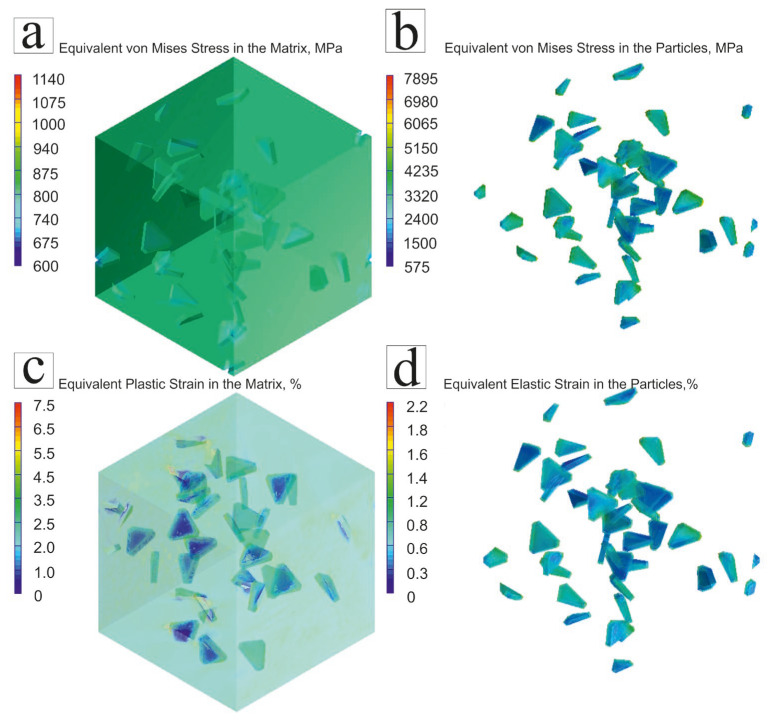
Equivalent stresses (MPa) between the Ti-4.25Al-2V matrix and SiC particles at 20 °C (**a**); equivalent stresses (MPa) in the transition-zone SiC particles at 20 °C (**b**); strain (%) distribution between the Ti-4.25Al-2V matrix and SiC particles at 20 °C (**c**); strain (%) distribution in the transition zone and SiC particles at 20 °C (**d**).

The results of the predictions (considering the matrix, transition zone, and ceramic particles) in terms of the stress and strain partitioning between the Ti-4.25Al-2V matrix and SiC particles at 20 °C are shown in Figure 4a,b and Figure 4c,d, respectively. The obtained results indicate the areas that can potentially fail under load. Specifically, Figure 4a,b show the transfer of the resulting stresses on the finite element grid from the matrix to the SiC particles. This finding is associated with a higher ductility and lower strength of the matrix in comparison with SiC particles, due to which the stresses arising in the matrix were transferred to the particles; the latter (depending on the shape, size, and distance between them) act as obstacles for deformation propagation. This phenomenon is well described by the theory of dispersion strengthening [32,33]. Moreover, it should be noted that most of the stresses are located in the transition zone and boundaries of the SiC particles, as indicated by the color code in Figure 4a,b. The deformation of the finite element grid of a representative volume of the material also suggests significantly lower strain levels of the SiC particles (the average value is about 0.8%) in comparison with those of the matrix (the average value is about 6%) (Figure 4c,d). Since the perfect contact between the nodes of the grid transition zone of the SiC particles and the matrix was used during the creation of the finite element model, the nodes of the matrix drag the nodes of the transition zone of the SiC particles, which causes deformation in the edge zones.

The obtained results show the efficacy of the Digimat-FE module in the prediction of some of the physical and mechanical properties of PRCMs at the micro level. Moreover, the interaction between the matrix and SiC particles can be visualized. In addition, the results of the prediction can be transferred to the modeling environment of the Explicit Dynamics module with which an improved accuracy in determining the mechanical properties such as tensile strength, plastic deformation, etc., can be attained (this is discussed further in Section 3.2).

Furthermore, similar modeling of the PRCM was carried out in temperatures of 250 °C, 350 °C, and 450 °C; the main strength characteristics were determined using the additivity rule [34]:

$$E=\varepsilon_{1}\varphi +(1-\varphi )\varepsilon_{2}$$

where E—the determined parameter, *ε*_1_—the parameter of the matrix material, *ε*_2_—the parameter of the reinforcing material, *φ*—the volume fraction of the matrix material, and (1 − *φ*)—the volume fraction of the reinforcing material. 

For further analysis, the obtained data are summarized in Table 2.

### 3.2. Numerical Modeling and Experimental Verification of Mechanical Behavior of PRCM

**Figure 5:**
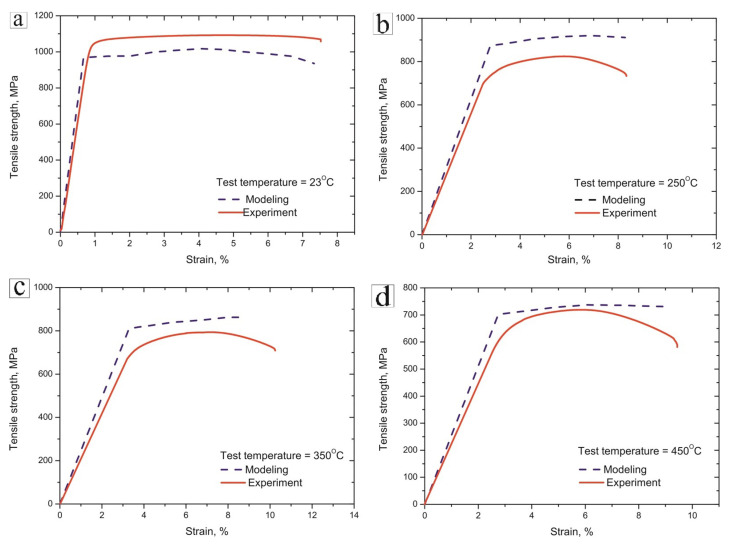
Stress–strain curves of PRCM samples (results of numerical modeling—dashed blue line; test results—solid red line): (**a**) at 23 °C; (**b**) 250 °C; (**c**) 350 °C; (**d**) 450 °C.

The numerical modeling of the mechanical behavior of the LDED samples was carried out in the Explicit Dynamics module using the finite element method via the AUTODYN. The results of the Explicit Dynamics simulation and comparison with the real mechanical behavior of the samples tested in tension are shown in Figure 5.

The presented curves clearly show that the numerical modeling using the Digimat Student Edition ver. 2022.4 and Ansys Student 2023 R2 software packages has an error of approximately 5–10%, which indicates that the algorithm is sufficiently correct to predict the mechanical characteristics of the PRCM. However, some differences in the predicted and experimental mechanical behavior can be observed. The predicted strength was slightly lower at room temperature and slightly higher at elevated temperatures in comparison with the experimental curves. Moreover, at elevated temperatures, the experimental curves suggest the formation of a neck at 4–5% elongation, while the numerical modeling suggests a nearly uniform elongation until the fracture (Figure 5b–d, Table 3). This phenomenon is associated with the imperfection of the finite element method used in modeling the experiment, since in order to obtain the most accurate data, it is necessary to break down the 3D model of the sample with the dimensions of the finite elements close to the dimensions of the crystal lattice of the material. To calculate such a model would require huge computing power and a significant amount of time, which would significantly complicate the entire process of modeling the sample.

The diagrams presented in Figure 5 clearly demonstrate that the PRCM’s digital tests have an error of approximately 5–8%, which is acceptable for numerical modeling. The imperfection of the finite element grid is the cause of the differences in the predicted and experimental results. Therefore, for the best computational results, it is necessary to divide the finite elements into smaller components (orders of 0.01 mm and less). This task is demanding and requires a significant amount of computing power. In this study, a finite element model was selected, which is optimal both for comparing the results with experimental data and for the resources used in the calculation.

The destruction of titanium alloys at elevated temperatures is due to the fact that when heated the metal becomes more ductile and less durable due to changes in the microstructure of the material, namely the changes in the size and shape of the metal grains and changes in the number and nature of defects within the material. It should be noted that the thermal effect on the material also contributes to an increase in the mobility of atoms in its crystal structure. This increase in mobility promotes the diffusion of atoms to defects in the material, which can lead to the destruction of atomic bonds and the weakening of the material as a whole. In addition, elevated temperatures contribute to the thermal relaxation of the material. This is a process in which internal stresses that have arisen as a result of various production operations or mechanical influences are reduced under the influence of temperature. As a result of such relaxation, microcracks may occur and increase, which negatively affects the mechanical properties of the material and reduces its service life [35].

**Figure 6:**
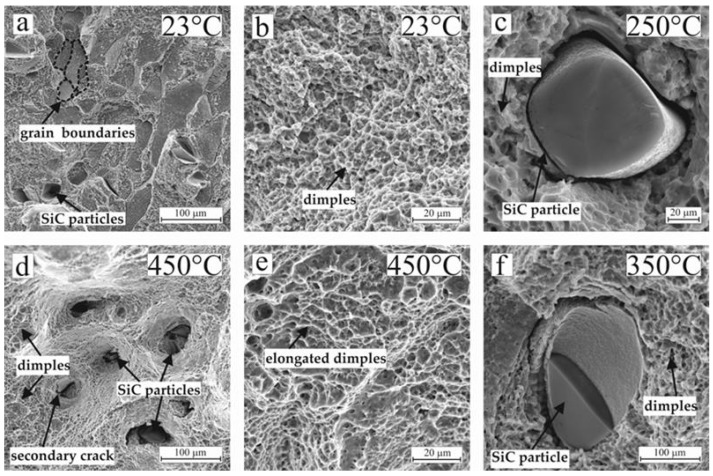
Fracture surface of PRCM samples: (**a**) fracture at 23 °C; (**b**) dimples at 23 °C; (**c**) SiC particle at 250 °C; (**d**) fracture at 450 °C; (**e**) dimples at 450 °C; (**f**) SiC particle at 350 °C.

Analyzing the fracture surface (Figure 6) obtained after tensile tests at different temperatures; a decrease in the strength of the material and increase in ductility can be observed. 

Figure 6 shows SEM images of the fracture surfaces of the LDED samples tested at different temperatures. The fracture of the sample tested at 23 °C is fibrous with a characteristic sub-grain fracture. The surface of all the fractures consists of many shallow dimples (a characteristic element of ductile fractures of titanium alloys [36]). Detailed fracture analysis revealed that large and smooth dimples are present in some areas, however, indicating the process of metal drawing into the areas free of particles (Figure 6a,d).

As the test temperature increases, a mixed fracture pattern is formed; in addition to the fibrous outer zone, an inner rounded zone with radial scars is present due to a more ductile behavior of the material at elevated temperatures. The fracture at elevated temperatures is characterized by rather large and deep dimples, indicating more pronounced plastic deformation, compared with samples tested at room temperature (Figure 6b,e).

On the surface of all the fractures, the SiC particles are randomly distributed. It can be suggested that cracks initiated in the SiC particles then propagate into the matrix. The bond between the SiC particles and the matrix appeared to be strong, however. High-magnification images of the fracture surface showed dimples surrounding the SiC particles, indicating that the matrix is fracturing through a ductile fracture mechanism (Figure 6c,f).

## 4. Conclusions

This study demonstrated the successful numerical modeling of the physical and mechanical properties of a dispersed–reinforced composite material (PRCM) using the Digimat 2022.4 and Ansys Student 2023 R2 software. It is possible to predict with sufficient accuracy the mechanical properties of the PRCM based on a near-α titanium alloy Ti-4.25Al-2V with 1 vol.% of ceramic SiC particles, which further allows the determination of the properties of the same composite materials based on different metal alloys and ceramic inclusions using similar programs. A simulation of the physical and mechanical properties was carried out for the PRCM samples of Ti-4.25Al-2V + 1 vol.% SiC by laser direct energy deposition. The numerical modeling results are consistent with the experimental data (within 10%).

It is shown that the addition of ceramic powder leads to an increase in the mechanical properties. With increasing test temperatures, the LDED samples became more plastic due to the plastic titanium matrix. The elongation of the material changed from 7.5% at room temperature to 11.6% at 450 °C, and with increasing temperature the tensile strength decreased from 1090 MPa to 720 MPa. However, by adding ceramic particles it is possible to achieve an insignificant loss of strength properties.

The algorithm for the prediction of the physical and thermomechanical properties of the PRCM based on titanium alloy Ti-4.25Al-2V with the addition of 1 vol.% SiC ceramic particles can be used as an intermediate stage in the design of new parts and structures with high weight.

## Figures and Tables

**Table 1:** Technological parameters for LDED.

Laser Power, W	Scan Speed, mm/s	Laser Beam Diameter, mm	Width Offset, mm	Height Offset, mm	Ti-4.25Al-2V Powder Feed Rate, g/min	SiC Powder Feed Rate, g/min	Argon Shielding Gas Flow (L/min)
1800	25	2.5	1.67	0.6	7.6	0.1	15

**Table 2:** Calculated values of PRCM properties using the Digimat Student Edition ver. 2022.4.

Value/ Temperature	20 °C	250 °C	350 °C	450 °C
Tensile strength, MPa	1029	868	806	698
Tensile strain, %	6.3	7.3	8.2	8.9
Poisson’s ratio	0.29	0.32	0.36	0.37

**Table 3:** Mechanical properties of samples at different test temperatures in comparison with the modeling results using Ansys Student 2023 R2 Explicit Dynamics module.

Test Temperature, °C	Yield Strength, MPa		Tensile Strength, MPa		Elongation, %	
	Modeling	Experiment	Modeling	Experiment	Modeling	Experiment
23	970	1050	1017	1090	6.6	7.5
250	870	710	919	809	7.6	9.6
350	730	687	850	782	8.0	10.5
450	700	617	735	720	9.1	11.6

## Data Availability

The data presented in this study are available on request from the corresponding author. The data are not publicly available because it is a part of an ongoing study.

## References

[1] Salunkhe S., Rajamani D. (2023). 3—Current trends of metal additive manufacturing in the defense, automobile, and aerospace industries. Advances in Metal Additive Manufacturing.

[2] Prathyusha A., Babu G.R. (2022). A review on additive manufacturing and topology optimization process for weight reduction studies in various industrial applications. Mater. Today Proc..

[3] Wrobel R., Scholes B., Hussein A., Law R., Mustaffar A., Reay D. (2020). A metal additively manufactured (MAM) heat exchanger for electric motor thermal control on a high-altitude solar aircraft—Experimental characterization. Therm. Sci. Eng. Prog..

[4] Badkoobeh F., Mostaan H., Rafiei M., Bakhsheshi-Rad H.R., RamaKrishna S., Chen X. (2023). Additive manufacturing of biodegradable magnesium-based materials: Design strategies, properties, and biomedical applications. J. Magnes. Alloys.

[5] Zhao N., Parthasarathy M., Patil S., Coates D., Myers K., Zhu H., Li W. (2023). Direct additive manufacturing of metal parts for automotive applications. J. Manuf. Syst..

[6] Gibson I., Rosen D., Stucker B. (2015). Additive Manufacturing Technologies: 3D Printing, Rapid Prototyping, and Direct Digital Manufacturing.

[7] Carroll B.E., Palmer T.A., Beese A.M. (2015). Anisotropic tensile behavior of Ti–6Al–4V components fabricated with directed energy deposition additive manufacturing. Acta Mater..

[8] Sun C., Song M., Wang Z., He Y. (2011). Effect of Particle Size on the Microstructures and Mechanical Properties of SiC-Reinforced Pure Aluminum Composites. J. Mater. Eng. Perform..

[9] Rao H., Oleksak R.P., Favara K., Harooni A., Dutta B., Maurice D. (2020). Behavior of yttria-stabilized zirconia (YSZ) during laser direct energy deposition of an Inconel 625-YSZ cermet. Addit. Manuf..

[10] Kovalenko E., Krasanov I., Valdaytseva E., Klimova-Korsmik O., Gushchina M. (2023). Influence of Laser Direct Energy Deposition Process Parameters on the Structure and Phase Composition of a High-Entropy Alloy FeCoNiCrMn. Metals.

[11] Kuzminova Y.O., Dubinin O.N., Gushchina M.O., Simonov A.P., Konev S.D., Sarkeeva A.A., Zhilyaev A.P., Evlashin S.A. (2023). The mechanical behavior of the Ti6Al4V/Ti/Ti6Al4V composite produced by directed energy deposition under impact loading. Materialia.

[12] Kumar R., Kumar M., Chohan J.S. (2021). The role of additive manufacturing for biomedical applications: A critical review. J. Manuf. Process..

[13] Promakhov V.V., Zhukov A.S., Vorozhtsov A.B., Schults N.A., Kovalchuk S.V., Kozhevnikov S.V., Olisov A.V., Klimenko V.A. (2019). Structure and Mechanical Properties of 3D-Printed Ceramic Samples. Russ. Phys. J..

[14] Maurya H., Jayaraj J., Vikram R., Juhani K., Sergejev F., Prashanth K. (2023). Additive manufacturing of TiC-based cermets: A detailed comparison with spark plasma sintered samples. J. Alloys Compd..

[15] Filippov A.A., Fomin V.M., Malikov A.G., Orishich A.M. (2016). Selective laser sintering of cermet mixtures Ti and B4C. AIP Conf. Proc..

[16] Li J., Yu Z., Wang H., Li M. (2012). Microstructure and Mechanical Properties of an in situ Synthesized TiB and TiC Reinforced Titanium Matrix Composite Coating. J. Wuhan Univ. Technol.-Mater. Sci. Ed..

[17] Jin G., Li Y., Cui H., Cui X., Cai Z. (2016). Microstructure and Tribological Properties of In Situ Synthesized TiN Reinforced Ni/Ti Alloy Clad Layer Prepared by Plasma Cladding Technique. J. Mater. Eng. Perform..

[18] Xi L., Ding K., Gu D., Guo S., Cao M., Zhuang J., Lin K., Okulov I., Sarac B., Eckert J. (2021). Interfacial structure and wear properties of selective laser melted Ti/(TiC+TiN) composites with high content of reinforcements. J. Alloys Compd..

[19] Krakhmalev P., Yadroitsev I. (2014). Microstructure and properties of intermetallic composite coatings fabricated by selective laser melting of Ti–SiC powder mixtures. Intermetallics.

[20] Li N., Xiong Y., Xiong H., Shi G., Blackburn J., Liu W., Qin R. (2019). Microstructure, formation mechanism and property characterization of Ti + SiC laser cladded coatings on Ti6Al4V alloy. Mater. Charact..

[21] Wang Z., Du J., Su G., Sun Y., Zhang C., Kong X. (2022). Microstructure, preparation and properties of TiC-Fe/FeCoCrNiMn cermet with a core-rim structure. Vacuum.

[22] Gan X., Xie D., Yang Q., Zhou K., Xiong H. (2019). Ti(C, N)-based cermets by vacuum sintering using Commercial Composite powders: Morphology evolution, Composition and related properties. Vacuum.

[23] Xing J., Du C., He X., Zhao Z., Zhang C., Li Y. (2019). Finite Element Study on the Impact Resistance of Laminated and Textile Composites. Polymers.

[24] Buryachenko V., Roy A., Lafdi K., Anderson K., Chellapilla S. (2005). Multi-scale mechanics of nanocomposites including interface: Experimental and numerical investigation. Compos. Sci. Technol..

[25] Yıldırım F., Demirel B., Bulucu E.D. (2022). Investigation of the mechanical properties of calcite reinforced polypropylene by using digimat-mean field homogenization and ansys FEM. Mater. Today Commun..

[26] Stasa F.L. (1985). Applied Finite Element Analysis for Engineers.

[27] Moaveni S. (2015). Finite Element Analysis: Theory and Application with ANSYS.

[28] (2021). Standard Test Methods for Elevated Temperature Tension Tests of Metallic Materials.

[29] Bistrova Y., Shirokina E., Mendagaliev R., Gushchina M., Unt A. (2019). Research of Mechanical Properties of Cold Resistant Steel 09CrNi2MoCu after Direct Laser Deposition. Key Eng. Mater..

[30] Shalnova S.A., Volosevich D.V., Sannikov M.I., Magidov I.S., Mikhaylovskiy K.V., Turichin G.A., Klimova-Korsmik O.G. (2022). Direct energy deposition of SiC reinforced Ti–6Al–4V metal matrix composites: Structure and mechanical properties. Ceram. Int..

[31] Li X., Zhang W. (2021). Interfacial reaction in SiCf/C/TiAl matrix composites. J. Mater. Res. Technol..

[32] Cahn R.W., Haasen P. (1983). Physical Metallurgy.

[33] Ansell G., Lenel F. (1960). Criteria for yielding of dispersion-strengthened alloys. Acta Metall..

[34] Bai X., Bessa M.A., Melro A.R., Camanho P.P., Guo L., Liu W.K. (2015). High-fidelity micro-scale modeling of the thermo-visco-plastic behavior of carbon fiber polymer matrix composites. Compos. Struct..

[35] Zhang J.-S. (2010). Diffusional Growth of Creep Cavities—High Temperature Deformation and Fracture of Materials.

[36] Ramosena L.A., Dzogbewu T.C., du Preez W. (2022). Direct Metal Laser Sintering of the Ti_6_Al_4_V Alloy from a Powder Blend. Materials.

